# Shortwave infrared spatial frequency domain imaging for non-invasive measurement of tissue and blood optical properties

**DOI:** 10.1117/1.JBO.27.6.066003

**Published:** 2022-06-17

**Authors:** Anahita Pilvar, Jorge Plutzky, Mark C. Pierce, Darren Roblyer

**Affiliations:** a Boston University, Department of Electrical and Computer Engineering, Boston, Massachusetts, United States; b Brigham and Women’s Hospital, Harvard Medical School, Department of Medicine, Boston, Massachusetts, United States; c Rutgers, The State University of New Jersey, Department of Biomedical Engineering, Piscataway, New Jersey, United States; d Boston University, Department of Biomedical Engineering, Boston, Massachusetts, United States

**Keywords:** : diffuse optics, shortwave infrared, scattering, two-layer model

## Abstract

**Significance:**

The shortwave infrared (SWIR) optical window (∼900 to 2000 nm) has attracted interest for deep tissue imaging due to the lower scattering of light. SWIR spatial frequency domain imaging (SWIR SFDI) provides wide-field tissue optical property measurements in this wavelength band. Key design and performance characteristics, such as portability, wavelength selection, measurement resolution, and the effect of skin have not yet been addressed for SWIR SFDI.

**Aim:**

To fabricate and characterize a SWIR SFDI system for clinical use.

**Approach:**

The optimal choice of wavelengths was identified based on optical property uncertainty estimates and imaging depth. A compact light-emitting diode-based dual wavelength SWIR SFDI system was fabricated. A two-layer inverse model was developed to account for the layered structure of skin. Performance was validated using tissue-simulating phantoms and *in-vivo* measurements from three healthy subjects.

**Results:**

The SWIR SFDI system had a $\mu_{s}^{\prime }$ resolution of at least $0.03\;{\mathrm{mm}}^{-1}$ at 880 nm and $0.02\;{\mathrm{mm}}^{-1}$ at 1100 nm. The two-layer inverse model reduced the error in deeper layer $\mu_{s}^{\prime }$ extractions by at least 24% in the phantom study. The two-layer model also increased the contrast between superficial vessels and the surrounding tissue for *in-vivo* measurements.

**Conclusion:**

The clinic-ready SWIR SFDI device is sensitive to small optical property alterations in diffuse media, provides enhanced accuracy in quantifying optical properties in the deeper layers in phantoms, and provided enhanced contrast of subcutaneous blood vessels.

## 1 Introduction

The shortwave infrared (SWIR) optical window (∼900 to 2000 nm) has recently attracted interest for deep tissue imaging due to the lower scattering of light compared to the visible (VIS: 400 to 700 nm) and near-infrared (NIR: 700 to 900 nm) wavelength bands.^1^ SWIR imaging has accelerated in recent years due to the increased availability of SWIR-sensitive detectors.^2^ The SWIR optical window has previously been explored for deep fluorescence imaging using NIR/SWIR fluorescent dyes for applications, such as targeted cancer imaging and tumor surgery guidance.^3^^–^^5^ Reflectance imaging in the SWIR optical window has been explored for determination of peripheral edema, skin burn, and bruise imaging, but the planar reflectance data cannot reliably predict the optical properties of the tissue.^6^^–^^8^ Techniques such as diffuse optical spectroscopy and spatial frequency domain imaging (SFDI) are widely used to measure tissue optical properties in the NIR region, namely, the absorption coefficient ($\mu_{a}$) and the reduced scattering coefficient ($\mu_{s}^{\prime }$). SFDI measurements in the SWIR may be advantageous over other SWIR imaging techniques owing to its ability for quantitative assessment of tissue and blood optical properties. Compared to SFDI measurements in the NIR, SWIR SFDI is potentially capable of imaging deeper in tissue.^9^^–^^11^ Additionally, SWIR SFDI is sensitive to water and lipids, which makes the technique valuable for applications, such as monitoring of volume status, tracking edema, and lipid measurements.

In our prior work, we developed a laser-based hyperspectral SWIR SFDI system with the ability to extract water and lipid concentrations in the tissue and blood of mice and humans.^12^ We demonstrated that SWIR SFDI could be used to monitor tissue edema during inflammation, lipid content in tumors, and brown fat content in mice. A key experiment in that work was the measurement of non-invasive blood lipids before and after a high-fat meal using measured optical properties through intact skin. While preliminary, this demonstration suggests that one important application of SWIR SFDI may be the non-invasive assessment of tissue and blood nutrients and metabolites. Potential examples include blood lipids (e.g., triglycerides and cholesterol) and glucose, metabolic parameters that are often measured as critical parameters in clinical management. Current approaches to such measurements through phlebotomy or other invasive techniques pose significant issues: higher costs including technicians and chemical laboratories, risk for both patients and technicians, generation of waste and its safe disposal. Moreover, the ability to track changes in these parameters regularly, conveniently and over time with high accuracy is an important unmet clinical need for patients with life-threatening diseases such as diabetes and cardiovascular disease.^13^^,^^14^ The potential for improved imaging depth with SWIR SFDI makes the measurement of subcutaneous tissue and blood a good fit for this technology but remains largely unexplored.

Our previously reported hyperspectral SWIR SFDI system^12^ suffered from low acquisition speed and immobility given the use of a tunable Ti-sapphire laser for sample illumination at wavelengths from 680 to 1300 nm. Additionally, the layered structure of skin during measurements of subcutaneous tissue and blood remained unaccounted for, likely leading to errors due to partial volume effects. These limitations, which represent important obstacles toward extending SWIR SFDI to clinical applications, are addressed in data presented here.

In this study, we first describe the design of a new, portable, dual-wavelength SWIR SFDI instrument for measuring the optical properties of subcutaneous tissue and blood. We identify the optimal imaging wavelengths based on an analysis of optical property uncertainties and imaging depth. Next, we show results obtained by using a new two-layer inverse model of the skin. We then report *in-vivo* measurements from three healthy subjects and show enhanced contrast of superficial blood vessels with the two-layer model.

## 2 SWIR SFDI Design Criteria

### 2.1 SFDI and SWIR Imaging Background

SFDI is a label-free, non-contact, diffuse optical imaging modality that provides measurements of optical properties of a biological sample ($\mu_{a}$ and $\mu_{s}^{\prime }$) from a large field of view on a pixel-by-pixel basis.^15^ SFDI often combines an optical source with a digital micro-mirror device (DMD) to project two-dimensional spatial patterns of light at multiple spatial frequencies and wavelengths onto the tissue. Typically, sinusoidal spatial patterns are projected on the sample with three phase offsets (0 deg, 120 deg, and 240 deg) at each wavelength and spatial frequency. The remitted light from the tissue, which contains the tissue response to the projected pattern, is captured by a CCD or CMOS camera. The captured images at each phase offset ($I_{1}$, $I_{2}$, $I_{3}$) are then demodulated using a demodulation algorithm [Eq. (1)] and calibrated using a measurement from a phantom with known optical properties to account for the instrument response

$$I=\frac{\sqrt{2}}{3}\sqrt{{\left(I_{1}-I_{2}\right)}^{2}+{\left(I_{2}-I_{3}\right)}^{2}+{\left(I_{1}-I_{3}\right)}^{2}.}$$ (1)

**Fig. 1:**
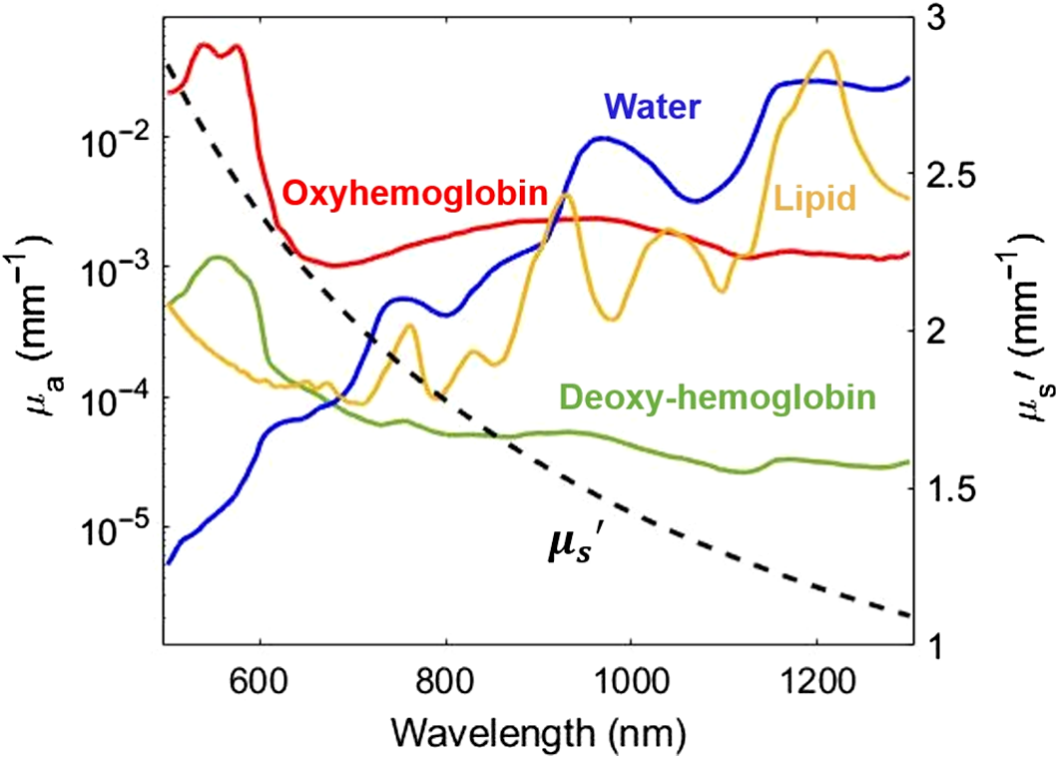
The absorption and reduced scattering of a representative human tissue from 500 to 1300 nm. Left y axis: the absorption coefficients of oxyhemoglobin (7.74  μM), deoxyhemoglobin (0.12  μM), water (21.42%) and lipid (27.74%) represent previously published measurements taken from human subjects on the dorsal forearm with skin types I and II.^16^ Left y axis shown in log-scale. $\mu_{a}$ was calculated by multiplying the extinction coefficient by the concentration for each chromophore. Right y axis: reduced scattering coefficient of human skin in linear scale.

This process generates images of the diffuse reflectance of the sample at each wavelength and spatial frequency. The diffuse reflectance values typically at two spatial frequencies are used as the inputs to an inverse model of light transport, which decouples and quantifies the relative contribution of absorption and reduced scattering at each wavelength. As a result, the calibrated diffuse reflectance measurements at two illuminating spatial frequencies are converted to the corresponding $\mu_{a}$ and $\mu_{s}^{\prime }$ values at each wavelength for every pixel in the field of view. SWIR SFDI has multiple potential advantages compared to VIS or NIR SFDI. First, the scattering of light generally decreases exponentially with wavelength in tissue at NIR and SWIR wavelengths, allowing for deeper light penetration at some SWIR wavelengths. This is especially true at wavelengths such as 1100 nm where the absorption from water, lipids, and collagen are at a local minimum.^9^^–^^11^ Second, the lower hemoglobin absorption and stronger absorption of water and lipids make measurements in the SWIR region more sensitive to water and lipid content. Figure 1 shows the absorption and reduced scattering coefficients of human skin tissue in VIS-NIR-SWIR optical windows, highlighting both the reduced scattering and prominence of water and lipid absorption at wavelengths past 900 nm.^16^

Below, we identify SFDI optimal spatial frequencies and wavelengths for sensitive and quantitative deep tissue imaging.

### 2.2 Wavelength and Spatial Frequency Selection

The choice of illumination wavelength and spatial frequency directly affects the uncertainties in calculated $\mu_{a}$ and $\mu_{s}^{\prime }$ values. In our previous work, we developed a method based on the Cramer Rao Bound (CRB) to estimate optical property uncertainties in SFDI based on the experimental measurement of diffuse reflectance uncertainties as function of wavelength, spatial frequency choice, and sample optical properties.^17^ Here, we used the CRB method to identify illumination wavelengths and spatial frequencies that minimize the uncertainties in optical property measurements in the SWIR spectral band.

We first established a diffuse reflectance error model for our previous hyperspectral SWIR SFDI system. Since this system can take measurements over a wide wavelength range, it provides a means to directly compare uncertainties across wavelengths. A drift test was conducted over 5 h from four intralipid samples with 2.5%, 5%, 7.5%, and 10% lipid concentrations.^12^ The SFDI measurements were taken every 30 min from each sample (10 measurements at each concentration). The measurements were taken at seven wavelengths from 700 to 1300 nm with 100-nm increments, and eight spatial frequencies from DC to $0.5\;{\mathrm{mm}}^{-1}$. The raw data at each wavelength and spatial frequency were converted to calibrated diffuse reflectance using the calibration measurement taken from 10% intralipid prior to the experiment. The error model was constructed by averaging the ratio of standard deviation to the mean value (at each wavelength and spatial frequency) over the measurements (Figure S1 in the Supplemental Material). The calculated noise model was then used as input for the CRB algorithm, which converted the diffuse reflectance uncertainties to uncertainties in estimated optical properties. A Monte Carlo (MC)-based look-up table (LUT) inversion algorithm was used for all analyses.

We first identified the spatial frequency pairs that provided the lowest uncertainties across the range of tested wavelengths and physiologically relevant optical properties. The uncertainty estimates for $\mu_{a}$ and $\mu_{s}^{\prime }$ were calculated for a wide set of spatial frequency pairs. Since uncertainties are also a function of sample optical properties, they were computed at five sets of previously measured optical properties that are representative of the dorsum of the hand.^12^ The minimum uncertainties occurred when DC ($0\;{\mathrm{mm}}^{-1}$) illumination was paired with an AC illumination of either 0.1, 0.15, or $0.2\;{\mathrm{mm}}^{-1}$, with only minor differences between these choices (Figure S2 in the Supplemental Material).

**Fig. 2:**
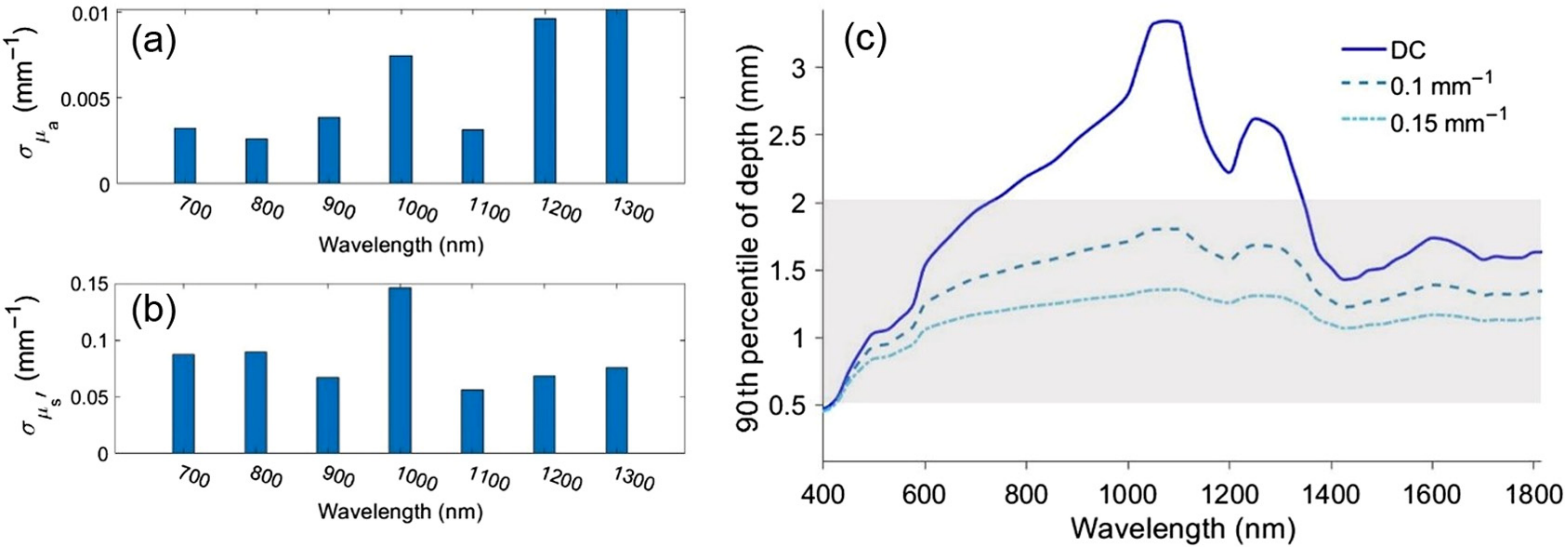
The CRB results for (a) $\mu_{a}$ and (b) $\mu_{s}^{\prime }$ uncertainties at a spatial frequency pair of DC and $0.15\;{\mathrm{mm}}^{-1}$. (c) Photon 90th percentile depth penetration for skin optical properties at three spatial frequencies (DC, 0.1 and $0.15\;{\mathrm{mm}}^{-1}$).^10^ The shaded area shows the reported range for human skin thickness.^18^^–^^20^

We then optimized the choice of illumination wavelengths to maximize the sensitivity of SFDI measurements to small changes in absorption and scattering properties of tissue and blood. Figures 2(a) and 2(b) show $\mu_{a}$ and $\mu_{s}^{\prime }$ uncertainties across seven wavelengths from 700 to 1300 nm when using the spatial frequency pair DC and $0.15\;{\mathrm{mm}}^{-1}$. The results show the smallest uncertainties in $\mu_{a}$ and $\mu_{s}^{\prime }$ at 800 and 1100 nm, respectively. Smaller theoretical uncertainties suggest higher sensitivity to small changes in optical properties compared to the other tested wavelengths. While this analysis identified wavelengths with the lowest uncertainties, for many applications it is also important to evaluate the sensitivity of a measured parameter at a specific wavelength to the underlying changes in tissue (e.g., chromophore concentration changes or scattering changes).

Additionally, depth penetration was considered as an important metric for wavelength selection. Sensitivity to optical properties of the subcutaneous tissue and superficial blood vessels underneath the skin requires measurements that probe deeper than the dermal layer of skin. The thickness of human skin has previously been reported to be in the range of 0.5 to 2 mm. We also sought to minimize the effect of highly absorbing chromophores present in skin epidermis layer, such as melanin, to have meaningful measurements in people with different skin tones.^21^

To estimate the imaging penetration depth we used a MC-based SFDI depth calculator reported in our prior work.^22^ Figure 2(c) shows the 90th percentile of photon penetration depth for skin from 400 to 1800 nm at spatial frequencies of DC, 0.1 and $0.15\;{\mathrm{mm}}^{-1}$.^10^ The plot shows a maximum penetration depth at ∼1100  nm, and also reveals that spatial frequencies up to $0.15\;{\mathrm{mm}}^{-1}$ pass through at least 1 mm of skin. The penetration depth results suggest that SFDI measurements taken at SWIR wavelengths and spatial frequencies of DC, 0.1 and $0.15\;{\mathrm{mm}}^{-1}$ are sensitive to the optical properties of the tissue and blood into the dermal layer, and in some cases, below the dermal layer.

The CRB analysis suggests the use of 800 and 1100 nm for minimizing the uncertainties in $\mu_{a}$ and $\mu_{s}^{\prime }$ extraction, respectively. These two wavelength are also in the spectral range where penetration depth is above 1 mm at spatial frequencies of DC to $0.15\;{\mathrm{mm}}^{-1}$ [Fig 2(c)]. The final choice of wavelength was made after searching through the available light-emitting diodes (LEDs) that provide high output power. While reviewing available parts from multiple suppliers, we ultimately chose Thorlabs LEDs, which are available over a wide wavelength range. The 880- and 1100-nm center wavelength LEDs from Thorlabs provide high output power while also satisfying the other two wavelength selection criteria.

## 3 SWIR SFDI System

### 3.1 System Hardware Design

**Fig. 3:**
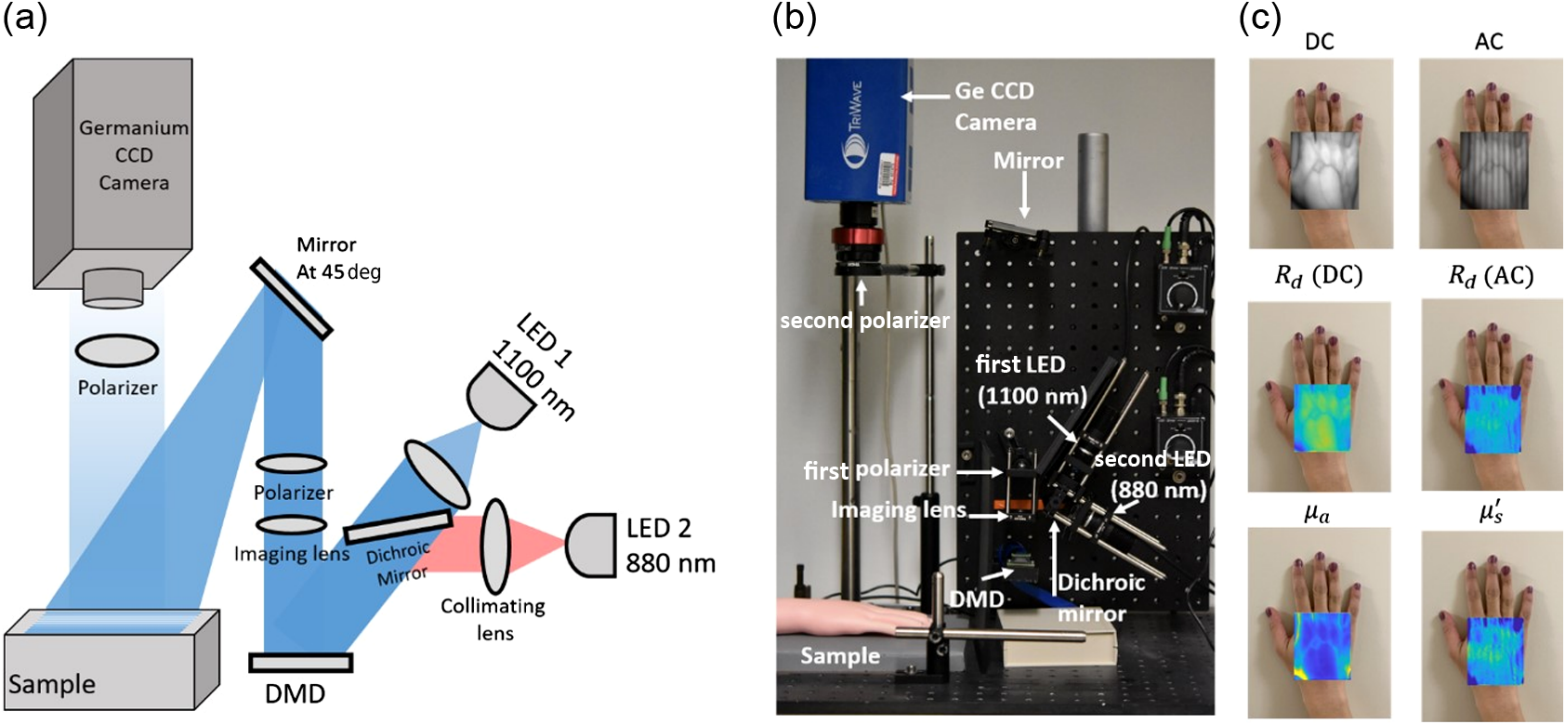
(a) Schematic diagram of the SWIR SFDI system. Light from the two LEDs is collimated and combined at a dichroic mirror. The DMD generates the spatially modulated light that projects onto the sample with an achromatic lens. Two linear polarizers are placed in the illumination and detection path to reject specular reflection. (b) The fabricated SWIR SFDI system. (c) Representative images from the system captured at 880 nm. Top row represents the raw images with DC and AC illumination patterns superimposed on the white light image of the hand taken with a camera phone. The middle row represents the calibrated diffuse reflectance at two spatial frequencies. Bottom row shows the optical property maps ($\mu_{a}$ and $\mu_{s}^{\prime }$).

A new LED-based SWIR SFDI system was designed and fabricated with the optimized wavelengths found in Sec. 2.2. The system utilizes two LEDs, one at 880 nm in the NIR (M880L3, Thorlabs), and one at 1100 nm in the SWIR (M1100L1, Thorlabs) with 50-nm FWHM for both LEDs. The LEDs provided ∼200 and 112 mW optical power at 880 and 1100 nm, respectively. The optical power at sample plane was $∼2.65\;\mu W\;{\mathrm{cm}}^{-2}$ at 880 nm and $3.1\;\mu W\;{\mathrm{cm}}^{-2}$ at 1100 nm. We followed the SFDI design steps that have been previously published by our lab and online in openSFDI.com and modified the design to build a compact and portable SFDI system.^23^ Figure 3(a) shows the SWIR SFDI system diagram. A long-pass dichroic mirror (DMLP1000, Thorlabs, cutoff wavelength=1000  nm) directs the collimated light (collimating lens: ACL25416U-B, Thorlabs) from both LEDs to the DMD (LC4500, Keynote Photonics). The pattern from the DMD was focused to the sample plane using an imaging lens (Ac254-050-B, Thorlabs). The projected light, which illuminates a large field of view (7×11  cm), is reflected from the sample and captured by a germanium CMOS camera (TriWave, Infrared Laboratories, Inc., Peabody, Massachusetts) with a wide spectral sensitivity (300 to 1600 nm). A SWIR imaging lens was used with the camera (SR0510-A01, StingRay, Keene, New Hampshire). A pair of crossed linear polarizers (LPNIRE100-B, Thorlabs) are placed in the illumination and detection paths to minimize the effect of specular reflection. The system is relatively fast (<1  min/whole sequence of DC+AC measurement, camera exposure time is 100 ms for each image at both wavelengths), compact and portable, making the system suitable for *in-vivo* and clinical measurements.

### 3.2 Calibration and In-Frame Phantom Correction

Demodulated DC and AC images were converted to diffuse reflectance images by measuring a phantom with known optical properties. A MC-based forward model of diffusive light propagation was used to extract the diffuse reflectance ($R_{d}$) of the calibration phantom based on its known optical properties. Here, we used a 10% intralipid suspension as the calibration phantom. The reduced scattering coefficient $\left[\mu_{s}^{\prime }=\mu_{s}(1-g)\right]$ of 10% intralipid suspension was adapted from Flock et al. Specifically, we assumed $\mu_{s}$ decreased exponentially with wavelength and the anisotropy factor (g) decreased linearly with wavelength per the equations provided in Flock et al.^24^ The absorption coefficient was calculated using pure water and lipid extinction spectra^25^^,^^26^ with Beer’s law. We ended up using a [$\mu_{a}\;\mu_{s}^{\prime }$] pair of $[0.0054\;3.64]\;{\mathrm{mm}}^{-1}$ at 880 nm and $[0.0181\;2.64]\;{\mathrm{mm}}^{-1}$ at 1100 nm for the calibration phantom. For the forward model used in the calibration process, intralipid was defined as a homogeneous semi-infinite medium with g and index of refraction (n) set to 0.7 and 1.33, respectively. These values were also adapted from Flock et al.^24^

A small 10% intralipid phantom (1×2×2  cm) was placed in the field of view as a gold standard phantom with known and stable optical properties. Assuming that the optical properties of the in-frame phantom do not change over time, we expect the $R_{d}$ values of the in-frame phantom to stay constant during measurements. For each measurement, the extracted $R_{d}$ values of the in-frame phantom were compared to the gold standard $R_{d}$ values (predicted by the MC model) at each wavelength and spatial frequency. Any discrepancy between the measured $R_{d}$ values and the gold standard values of the in-frame phantom translates into a correction factor and were used to correct the $R_{d}$ values of the entire field of view. This method was described in our prior work.^27^

### 3.3 System Characterization

To characterize the precision of the system, a repeat test measurement was performed by taking 10 back-to-back measurements of a 10% intralipid suspension at $f_{x}=\mathrm{DC}$ and $0.15\;{\mathrm{mm}}^{-1}$. The measured optical properties over the region of interest spanning the intralipid surface (∼2×2  cm, 14,400 pixels) were averaged and the standard deviation of the mean values over these ten measurements were calculated. The coefficients of variation of the repeat measurements for $\mu_{a}$ were 1.55% and 0.80% for 880 and 1100 nm (standard deviation $5.4\times 10^{-5}{\;\mathrm{mm}}^{-1}$ and $1.31\times 10^{-4}\;{\mathrm{mm}}^{-1}$). These values for $\mu_{s}^{\prime }$ were 0.20% and 0.21% for 880 and 1100 nm, respectively (standard deviation 0.0074 and $0.0057\;{\mathrm{mm}}^{-1}$).

We also calculated the theoretical precision of the system by conducting the CRB analysis with the noise model of the dual-LED system. The dual-LED system outperforms the laser-based system (smaller noise and higher precision), and the spatial frequency of 0.1 and $0.15\;{\mathrm{mm}}^{-1}$ paired with DC appears to be the optimized choice with the new system as well.

**Fig. 4:**
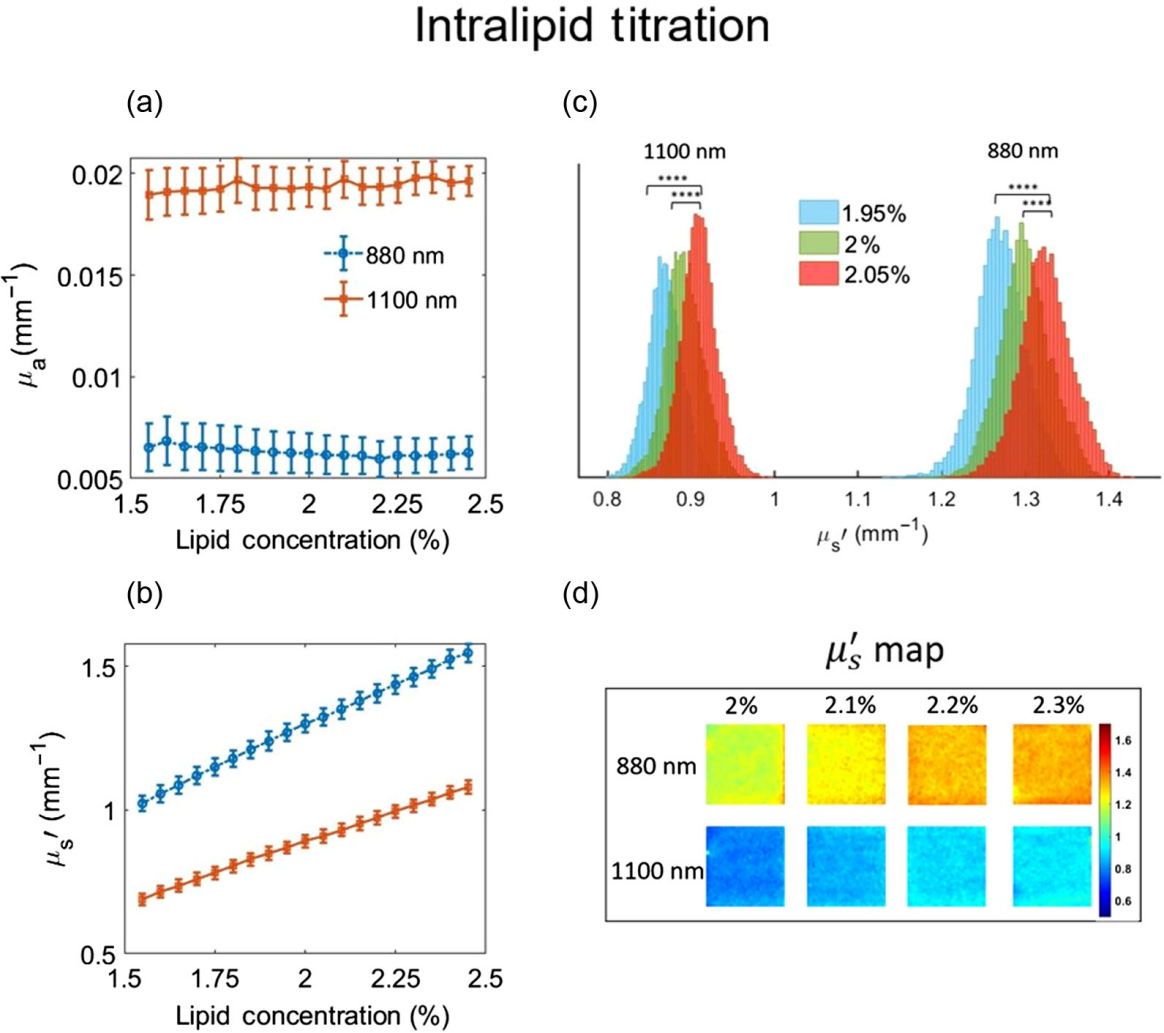
Results of an intralipid titration experiment to characterize the measurement sensitivity of SWIR SFDI to lipid concentration increments of 0.05% at 880 and 1100 nm. (a) The measured absorption coefficient stayed largely unchanged when lipid concentrations were increased from 1.55% to 2.45%. (b) The measured reduced scattering coefficient increased with increasing intralipid concentrations. (c) Histograms of $\mu_{s}^{\prime }$ pixel values from three consecutive measurements in the lipid titration experiment. The differences between the distribution of measurements of 1.95%, 2%, and 2.05% lipid concentration were statistically significant (**** indicates p<0.0001, determined with Student’s t-test). (d) The $\mu_{s}^{\prime }$ maps of four intralipid suspensions from 2% to 2.3% concentrations. All four concentrations were placed in the same field of view and measured simultaneously.

To characterize the measurement resolution of $\mu_{s}^{\prime }$ extractions, intralipid samples with 19 different concentrations ranging from 1.55% to 2.45% with 0.05% increments were measured. Figure 4 shows the mean and standard deviation of the extracted optical properties from these measurements. The absorption remained almost unchanged over the different intralipid concentrations [Fig. 4(a)], while the reduced scattering increased gradually with increasing lipid concentrations [Fig. 4(b)]. Figure 4(c) shows the histogram of $\mu_{s}^{\prime }$ values for three different intralipid concentrations at 0.05% increments. Changes as small as 0.05% in lipid concentration resulted in statistically significant alterations in measured $\mu_{s}^{\prime }$ values (p<0.0001 using pairwise Student’s t-test). The 0.05% lipid increment translates to a $\mu_{s}^{\prime }$ increase of $∼0.03\;{\mathrm{mm}}^{-1}$ at 880 nm and $∼0.02\;{\mathrm{mm}}^{-1}$ at 1100 nm, indicating the $\mu_{s}^{\prime }$ resolution of the system is at least this small for the imaging parameters utilized. Figure 4(d) shows the imaging contrast in $\mu_{s}^{\prime }$ of four intralipid suspensions from 2% to 2.3% at 880 and 1100 nm.

We used the results from the intralipid titration experiment to evaluate the accuracy of the optical property extraction. We compared the measured $\mu_{a}$ values to the ground truth values calculated using Beer’s law. The SWIR SFDI results show on average 9.65% error ($0.00055\;{\mathrm{mm}}^{-1}$ absolute error) in extracted $\mu_{a}$ at 880 nm and 1.30% error ($-0.00008\;{\mathrm{mm}}^{-1}$ absolute error) in extracted $\mu_{a}$ at 1100 nm. For $\mu_{s}^{\prime }$ we expect to see a linear increase in $\mu_{s}^{\prime }$ with increasing lipid concentration. Going from 1.55% to 2.45% lipid concentration, we expect to see a 1.58-fold increase in $\mu_{s}^{\prime }$. The measured $\mu_{s}^{\prime }$ values show 1.56-fold and 1.50-fold increase at 880 and 1100 nm, respectively, closely matching expectations.

## 4 Two-Layer Model

Here, we developed a two-layer inverse model to better extract the optical properties of subcutaneous tissue and blood. Our group has previously developed a MC based two-layer LUT inversion model that accounts for the effect of mouse skin during preclinical tumor SFDI imaging in the NIR.^28^ In this prior work, the two-layer LUT reduced the errors in optical property extraction from the second, deeper layer in both a two-layer phantom study and in an *in-vivo* mouse study. Here, we used similar methods to account for the effect of human skin properties on the optical properties extracted from subcutaneous tissue and blood. The Gardner MC method, which obtains $R_{d}$ estimates natively in the spatial frequency domain, was used to generate the two-layer LUT.^29^ The top layer represents the human skin layer with fixed optical properties and thickness. The bottom layer is a semi-infinite geometry with optical properties as free parameters of the inversion algorithm. MC simulations were conducted using the Boston University shared computer cluster. We launched $10^{7}\text{ photons}$ for each simulation and a total of 160 simulations were conducted (one for each $\mu_{s}^{\prime }$ value). $\mu_{a}$ was set to $0.0005\;{\mathrm{mm}}^{-1}$ for the bottom layer for MC simulations. The results from each simulation were then post processed using Beer’s law to achieve the bottom layer $\mu_{a}$ range for the two-layer LUTs.

A phantom validation study was conducted to evaluate the effect of using the two-layer model to perform optical property extractions. We first used 5% intralipid to fabricate a skin mimicking phantom with optical properties similar to human skin in the 1000- to 2200-nm wavelength range.^30^ The phantom was made by mixing intralipid with agar powder and heating the mixture to form a solution, which formed a solid phantom after cooling. The optical properties of the phantom were measured using the SWIR SFDI system. We used the average optical properties of the phantom measured at 880 and 1100 nm to set the top layer $\mu_{a}$ and $\mu_{s}^{\prime }$ values to 0.004 and $0.88\;{\mathrm{mm}}^{-1}$, respectively. The anisotropy factor and refractive index were set to 0.7 and 1.33 for both top and bottom layers to match the properties of intralipid.^24^ The average thickness of human skin was reported as 1 mm and was set as a fixed parameter in MC simulations.^18^^,^^19^

**Fig. 5:**
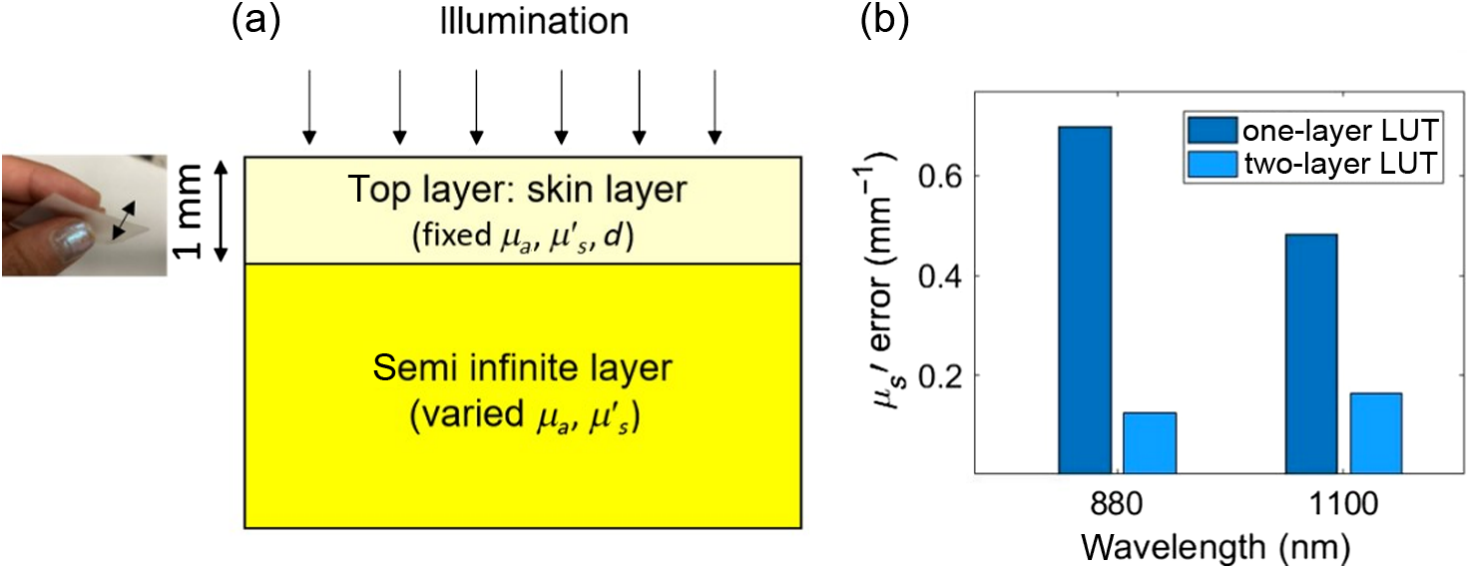
(a) A schematic of the two-layer MC simulation geometry. The inset shows a 1-mm thick skin-simulating phantom made with intralipid and agar. (b) Comparison of bottom layer $\mu_{s}^{\prime }$ measured using one-layer and two-layer LUTs. The absolute extraction errors for $\mu_{s}^{\prime }$ are shown.

A set of two-layer phantoms were fabricated to validate the two-layer LUT inverse model with the top-layer mimicking the skin tissue thickness (1 mm) and optical properties using 5% intralipid solid phantoms. The skin-mimicking phantoms were placed on top of the bottom liquid phantoms that consist of four intralipid suspensions from 2% to 5% lipid concentrations. SFDI measurements were taken from the two-layer phantoms and processed using both one-layer and the new two-layer LUT. Figure 5(a) shows the geometry of the two-layer MC simulations as well as a picture of a 1 mm thick skin phantom used in the phantom study. Figure 5(b) shows the average error in extracted $\mu_{s}^{\prime }$ from the bottom layer from the four intralipid suspensions. The two-layer model improves the accuracy of bottom layer $\mu_{s}^{\prime }$ extraction by 33.9% and 24.5% for 880 and 1100 nm, respectively. The two-layer LUT however does not improve the error in the extracted $\mu_{a}$, and errors for both one-layer and two-layer LUTs were ∼50% at both wavelengths, although this corresponds to a small absolute error of $∼0.005\;{\mathrm{mm}}^{-1}$.

The parameters of the two-layer LUT were then modified for human measurements. A second two-layer LUT was generated with a g value of 0.9 for both the skin layer and the semi-infinite bottom layer. The refractive index was set to 1.37 for the skin layer^10^^,^^30^ and 1.4 for the bottom layer. The $\mu_{a}$ value for the skin layer was set to $0.024\;{\mathrm{mm}}^{-1}$.^10^ The $\mu_{s}^{\prime }$ of the skin layer was set to $0.88\;{\mathrm{mm}}^{-1}$, which matches the $\mu_{s}^{\prime }$ of the LUT used in the phantom study and lies in the range reported for skin at 1100 nm.^10^^,^^31^ A third two-layer LUT was generated using $\mu_{s}^{\prime }$ of $1.25\;{\mathrm{mm}}^{-1}$, which matches the optical properties of skin at 880 nm.^31^^,^^32^ The same $\mu_{a}$ value of $0.024\;{\mathrm{mm}}^{-1}$ was used for both of these two-layer LUTs.

For the newly generated two-layer LUT, an additional round of CRB analysis was conducted for spatial frequency selection. While the prior analysis for single layer media indicated that DC illumination paired with an AC spatial frequency of 0.1, 0.15, or $0.2\;{\mathrm{mm}}^{-1}$ had approximately equivalent performance, the two-layer analysis indicated that DC and $0.1\;{\mathrm{mm}}^{-1}$ had superior performance compared to the other choices when attempting to extract the deeper layer optical properties. This is likely due to the deeper imaging depth of $0.1\;{\mathrm{mm}}^{-1}$ versus 0.15 or $0.2\;{\mathrm{mm}}^{-1}$ (Fig. 2). Subsequent human measurements were collected using $f_{x}=\mathrm{DC}$ and $0.1{\;\mathrm{mm}}^{-1}$.

We note that we also tried the model with g=0.3 which is closer to the reported g value in Ref. 33 for intralipid. The different models have a relative bias of 0.0009 and $0.0037\;{\mathrm{mm}}^{-1}$ in $\mu_{a}$, and 0.34 and $0.26\;{\mathrm{mm}}^{-1}$ in $\mu_{s}^{\prime }$ at 880 and 1100 nm respectively, but the choice of model with different g value did not affect the performance characteristic of the system and the performance of the two-layer model.

## 5 In-Vivo Blood and Tissue Measurements

**Fig. 6:**
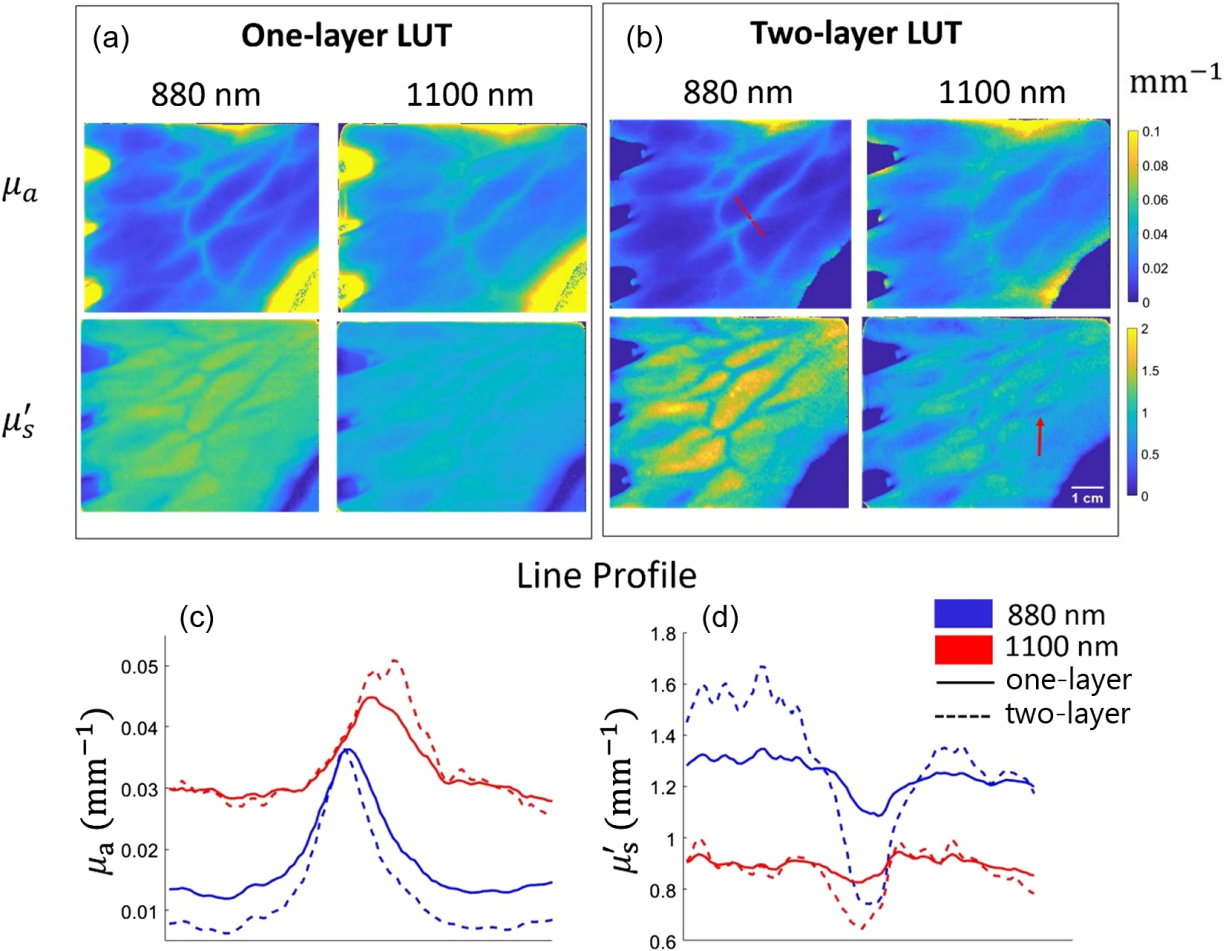
Healthy volunteer results (subject #1) from the dorsum of the hand. Optical property maps at 880 and 1100 nm using (a) one-layer LUT and (b) two-layer LUTs. Line profiles over the dotted red line for (c) $\mu_{a}$ and (d) $\mu_{s}^{\prime }$. Blue shows the line profiles at 880 nm and red shows the line profiles at 1100 nm. The solid lines correspond to the one-layer LUT results and dashed lines correspond to the two-layer LUT results. The use of the two-layer LUT enhances the contrast of the superficial blood vessel especially in $\mu_{s}^{\prime }$. The red arrow in (b) shows an example of a vessel-like structure that is identified in the $\mu_{s}^{\prime }$ map but not in the $\mu_{a}$ map at the same wavelength.

SWIR SFDI measurements were performed on the dorsal surface of the hand of three subjects: one 28-year-old female and two 28-year-old male subjects. The measurements were conducted under an institutionally-approved protocol (protocol number 4698). Subjects were informed about the study and provided informed consent prior to the experiment. The optical properties extracted when using the one-layer and two-layer LUTs were compared over large superficial vessel regions (dorsal metacarpal veins in the hand) and the surrounding tissue. The results from using each LUT were compared in terms of the contrast between the optical properties of superficial vessels and the surrounding tissue using line profiles. In Figs. 6(a) and 6(b), optical property maps for the dorsum of the hand at 880 and 1100 nm are shown for subject #1 using the one-layer LUT and the two-layer LUTs. The line profiles are plotted over the red dashed line in Fig. 6(b) for both $\mu_{a}$ and $\mu_{s}^{\prime }$ [Figs. 6(c) and 6(d)]. The contrast between the metacarpal veins and the surrounding tissue on the hand increases for both $\mu_{a}$ and $\mu_{s}^{\prime }$ when using the two-layer LUT compared to the one-layer LUT for all volunteers. We calculated the contrast over the line profile using the following equation (Weber contrast) at each wavelength and optical property (μ referrers to scattering or absorption coefficient):

$$C=\frac{\mu_{\max}-\mu_{\min}}{\mu_{\min}}.$$

On average, we saw a 352% and 223% increase in $\mu_{s}^{\prime }$ contrast and 104% and 61% increase in $\mu_{a}$ contrast at 880 and 1100 nm, respectively, averaged across all subjects over a designated superficial vessel on the hand. Table 1 summarize the contrast ratio (shown as percentages) between a superficial vessel and its surrounding tissue at both wavelengths and optical properties using one-layer and two-layer LUTs for all subjects. These results suggest that two-layer LUTs provide increased sensitivity to the large superficial blood vessels. We noted that some image features, e.g., vessels could be identified in the $\mu_{s}^{\prime }$ map while they cannot be identified in the corresponding $\mu_{a}$ map. One example is designated by the red arrow in Fig. 6(b). We note that the optical property maps and the contrast values represent the bottom layer when using the two-layer LUTs.

**Table 1:** The vessel-to-surrounding tissue contrast ratio over a metacarpal vein on the hand for three subjects using one-layer and two-layer LUTs.

	Subject #1		Subject #2		Subject #3	
	$\mu_{a}$	$\mu_{s}^{\prime }$	$\mu_{a}$	$\mu_{s}^{\prime }$	$\mu_{a}$	$\mu_{s}^{\prime }$
880 nm						
One-layer LUT	174%	20%	69%	17%	69%	16%
Two-layer LUT	407%	97%	124%	80%	137%	64%
1100 nm						
One-layer LUT	52%	13%	51%	18%	26%	15%
Two-layer LUT	85%	43%	86%	66%	39%	41%

## 6 Summary and Discussion

In this work, we have designed and validated a new dual-LED SWIR SFDI system for non-invasive measurement of tissue and blood optical properties. We identified optimal wavelengths and spatial frequencies for accurate estimation of optical properties, and we developed a two-layer model to improve the sensitivity of SFDI measurements to subcutaneous tissue and blood. The portable SWIR SFDI system was fabricated using two LEDs at 880 and 1100 nm. The spatial frequency pairs of DC-0.15 and DC-0.1 ${\mathrm{mm}}^{-1}$ were used for homogenous phantom and two-layer phantom/*in-vivo* measurements, respectively. The new SWIR SFDI system is fast (<1  min measurement time for two-wavelength measurements), compact and portable, which are distinct advantages compared to our previous hyperspectral SWIR SFDI system, which took approximately 2 min and 15 s for two-wavelength measurements. The new system is sensitive to small optical property alterations with $\mu_{s}^{\prime }$ resolution at least $0.03\;{\mathrm{mm}}^{-1}$ at 880 nm and at least $0.02\;{\mathrm{mm}}^{-1}$ at 1100 nm.

The two-layer model developed here decreased errors by at least 24% in the extracted bottom layer $\mu_{s}^{\prime }$ compared to the homogenous model in the two-layer phantom study. However, it did not result in an improvement in bottom layer $\mu_{a}$ estimation. Similarly, the estimated $\mu_{a}$ in the *in-vivo* measurements were less affected by the use of the two-layer inverse model compared to the estimated $\mu_{s}^{\prime }$. One possible explanation is the similarity between top and bottom layer $\mu_{a}$ in the phantom study. We used 5% intralipid suspension to fabricate the skin phantoms and the bottom layer consisted of four intralipid suspensions with 2% to 5% lipid concentrations. The $\mu_{a}$ of intralipid at 880 and 1100 nm is minimally affected by the alteration in lipid concentrations over the 2% to 5% range ($\mu_{a}$ (at 880 nm) = 0.0056 to $0.0057\;{\mathrm{mm}}^{-1}$ and $\mu_{a}$ (at 1100 nm) = 0.0189 to $0.0195\;{\mathrm{mm}}^{-1}$ for 2% to 5% lipid concentrations). Since both layers had similar $\mu_{a}$ values, the error in the extracted $\mu_{a}$ was minimally affected when using either the one-layer or two-layer LUT.

The use of the two-layer model revealed higher contrast between superficial vessels and their surrounding tissue compared to the homogeneous, one-layer model for *in-vivo* measurements of the hand. The optical absorption of blood is known to be substantially higher relative to skin and optical scattering of blood is often reported to be lower relative to skin.^34^^,^^35^ The higher contrast between superficial vessels and the surrounding tissue at both $\mu_{a}$ and $\mu_{s}^{\prime }$ can be interpreted as an improved sensitivity to optical properties of blood (i.e., $\mu_{a}$ increased more dramatically over vessels and $\mu_{s}^{\prime }$ decreased more dramatically over vessels compared to the single-layer LUT results). The optical properties of the tissue surrounding the superficial vessels stayed relatively unchanged when using either the single or two-layer LUTs for 1100 nm, but changed more substantially for 880 nm. This is possibly because the two-layer model helped to account for the effect of melanin absorption in the epidermis on the extracted $\mu_{a}$ at this wavelength, and it is known that melanin absorption is stronger at 880 nm compared to 1100 nm.^36^

SWIR SFDI suffers from a few important limitations. First, SFDI measurements are limited by a partial volume effect when measurements are taken on a heterogeneous media. In this work, we attempted to mitigate the effect of skin on subcutaneous optical property extraction by using a two-layer inverse model. Here, fixed optical properties and thickness for the skin were assumed in our model, which may reduce applicability to different body locations with different skin thickness and different skin tones; such adjustments however can be made for different measurement locations. Although SWIR wavelengths benefit from lower melanin absorption compared to the VIS-NIR optical window, people with different skin tones may have distinct skin optical properties.^16^^,^^36^ Further study is required to investigate the effect of the two-layer model on optical property extraction from subjects with different skin tones. Additionally, while the dual-LED SWIR SFDI system developed here has distinct advantages for clinical use, including being label-free, non-contact, with a relatively high acquisition speed, the fabrication cost is high compared to other VIS/NIR SFDI systems due to the high cost of the SWIR camera (currently scientific grade InGaAs SWIR cameras can cost well over $10k USD).

In summary, we have developed a clinic ready dual-LED SWIR SFDI system. We have generated a new two-layer inverse model that accounts for the layered effect of skin, validated this system and the model through measurements in phantom models as well as the hands of three healthy volunteers. Although additional development and study are needed, this SWIR SFDI system can measure the optical properties of tissue and blood. As such, this technology has the potential to provide a powerful novel tool for multiple clinical applications in a safe, cost effective, and efficacious manner for conditions such as cardiovascular diseases and diabetes.

## Code, Data, and Materials Availability

All relevant code, data, and materials are available from the authors upon reasonable request. Correspondence and requests should be addressed to the corresponding author.
