# Supplementary material for: Shortwave infrared spatial frequency domain imaging for non-invasive measurement of tissue and blood optical properties
Source: J Biomed Opt. 2022 Jun 17;27(6):066003. doi: 10.1117/1.JBO.27.6.066003 (PMC9204261; doi:10.1117/1.JBO.27.6.066003)
Supplement: Supplementary file 1 [file JBO_027_066003_SD001.pdf]

Supplementary Material

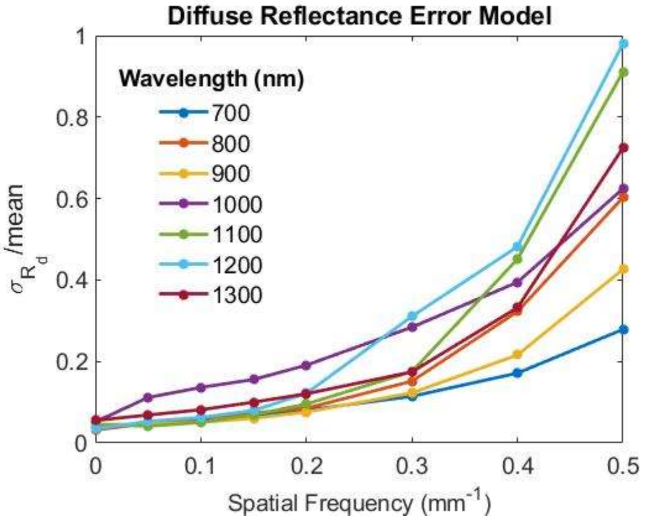

Fig S1. SWIR-MPI (meso-patterned imaging) noise model at seven wavelengths and eight spatial frequencies

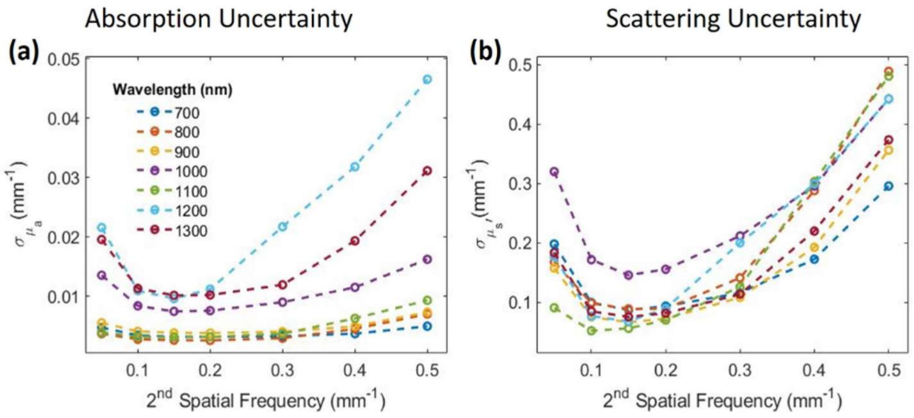

Wavelength (nm)	$\mu_a(\mathrm{mm}^{-1})$	$\mu_s'(mm^{-1})$
700	0.0197	1.4186
800	0.0178	1.3151
900	0.0221	1.2194
1000	0.0374	1.0891
1100	0.0185	1.0104
1200	0.0939	0.9031
1300	0.0694	0.8700

Fig S2. Cramer-Rao bound uncertainties at seven wavelengths for optical properties representative of the dorsum of the hand. A) Absorption uncertainty and b) reduced scattering uncertainty for a spatial frequency paired with DC.
